# Evaluation of  premetastatic changes in lymph nodes(pN0) of oral tongue tumour: A prospective observational Study

**DOI:** 10.12688/f1000research.138951.1

**Published:** 2023-07-26

**Authors:** Rajalakshmi Geetha, Subramania Iyer, Pavithran Keechilat, Gopalakrishna Iyer N, Krishna Kumar Thankappan, Smitha N V

**Affiliations:** 1 Head and Neck Surgery/Oncology, Amrita Institute of Medical Sciences - Amrita Vishwa Vidyapeetham, Kochi, Kerala, India; 2 Medical Oncology, Amrita Institute of Medical Sciences - Amrita Vishwa Vidyapeetham, Kochi, Kerala, India; 3 Head and Neck Surgery, National Cancer Centre Singapore, Singapore, Singapore; 4 Department of Pathology, Amrita Institute of Medical Sciences -Amrita Vishwa Vidyapeetham, Kochi, India

**Keywords:** Oral Tongue Squamous Cell Carcinoma, Premetastatic Niche, Circulating Tumor Cells, Circulating Tumor Emboli, Disease free survival.

## Abstract

**Background: **Tongue tumors show intra and inter-tumoral heterogenicity with high incidence, relapse and mortality rates necessitating further research.  Recurrence/metastasis that occurs  after surgical resection of primary cancer is often the reason for poor survival in these patients.  Lymph nodes are the most common site of metastasis in tongue tumors. Therefore, premetastatic molecular changes can be best evaluated in lymph nodes which may epitomize the earliest events in the metastasis cascades. The presence of circulating tumor cells(CTCs) in the absence of nodal disease (N0) may represent tumor aggressiveness, suggesting an immune escape which may have high metastatic potential. This trial  was developed  to investigate the earliest pre-metastatic changes which may regulate tumor dormancy and predict metastasis. A better understanding of organotropism or pre-metastatic changes can help in theragnostic, thereby  preventing the outbreak of overt metastasis.

**Methods:** A single-institutional prospective observational cohort study. This trial will be conducted at a tertiary care Centre (Amrita Institute of Medical Sciences Kochi).  Eligible patients will be enrolled after obtaining informed consent. The dissected lymph nodes will  be subjected to histopathological and immunohistochemical analyses for premetastatic niche (PMN) formation. In addition, circulating tumor cells will be evaluated before treatment and 6 months after treatment. The patients will be followed  up for a period of two years to correlate the findings with the recurrence-free survival.

**Expected results:**  The pre-metastatic changes, if detected will  be  a predictive biomarker. It may help to define future drug targets for metastasis chemoprevention   . CTCs may  define the tumor aggressiveness ,there by  prognostication  and helps in better disease management.

**Ethics and dissemination:** The study has received the following approval:

Ethics Committee of Amrita School of Medicine (ECASM-AIMS-2022-048).Trial Registered Prospectively( CTRI/2022/03/041256 ) on 22/03/2022 under Clinical Trial Registry of India

**Abbreviations**
CK8/18/19: Cytokeratin 8/18/19
CTC: Circulating Tumour Cells
CTM: Circulating Tumour Micro emboli
DAMP: Damage-associated molecular pattern
ECS: Extra capsular Spread
EMT: Epithelial -Mesenchymal Transition
EpCAM: Epithelial cell adhesion molecule
FFPE: Formalin fixed -paraffin embedded
IHC: Immuno Histochemistry
LN: Lymphnode
LOX 2: Lysyl Oxidase 2
MDSCs: Myeloid-derived suppressor cells
OSCC: Oral Squamous Cell Carcinoma
OTSCC: Oral Tongue Squamous Cell Carcinoma
PMN: Premetastatic Niche
STAT 3: Signal Transducer and activator of transcription3
VEGF A: Vascular Endothelial Growth Factor A

## Introduction

Tumor metastasis is a major factor that leads to treatment failure and mortality. The cure for cancer metastasis is still challenging, so treating cancer effectively depend on our ability in arresting or preventing metastasis. Tumor mortality is a result of late diagnosis, so research into early detection is of greater importance in improving patient outcomes.

The WHO anticipates that by 2040, the global cancer burden will reach 27.5 million with approximately 16.3 million deaths. ^1^^,^^2^ In the WHO 2022 updates, oral squamous cell carcinoma is the 16 ^th^ most common cancer globally, and the incidence of tongue cancer in persons younger than 45 years has increased worldwide. ^3^ One-third global burden of oral cancer is from India, tongue and floor of mouth comprise more than 50% of oral cancers. ^4^ The high tongue tumor prevalence in India demands scaling up relevant research in this area to deliver an optimal outcome. Recent data projecting a distressing trend in the recurrence and mortality rates of tongue cancers with increasing incidence in the non-habit associated tongue tumor. ^5^

Despite of advances in treatment modalities, the inability to control the metastatic process is one of the common reasons for treatment failures and high morbidity rates. Tumor metastasis is still not fully understood. Research may be of great importance to understand the tumor biology, heterogenicity, disease presentation and progression for tailoring accurate, affordable early detection tools and disease monitoring to interdict metastasis, thus survival outcomes.

Our primary objective of this study is the molecular profiling of tumor-free lymph nodes (N0) to assess the PMN changes and correlate them with recurrence -free survival in oral tongue squamous cell carcinoma patients.

The secondary objective is to evaluate the efficacy of CTC (circulating tumor cells) and CTM (circulating tumor micro emboli) in terms of relapse, and disease-free survival in N0 patients.

### Rationale of study

Tongue tumors are a matter of concern for oncologists, researchers and public health policymakers. Patients with tongue tumors have a higher proportion of treatment failures, even after standard treatment protocols. Treatment and clinical decisions on N0 neck in the early stages are still challenging. The primary goal is to investigate the early pre metastatic alterations even before a detectable metastasis and its association in initiating metastasis.

This study may help in

• Elucidating molecular metastatic driving events before or during early stages of metastatic colonization.
• Identifying definite lymph node architecture parameters in predicting metastasis before tumor cells arrive.
• Studying the significance of CTC/CTM occurring at early nodal-free tumors.

## Methods

### PMN trial design

The PMN study is designed as a prospective cohort observational study.

### Study settings

This study will take place at the Tertiary Care Advanced Centre (Department of Head & Neck Oncology & Pathology), Amrita Institute of Medical Sciences, Kochi (India).

### Trial registration

PMN CTC trial protocol registration has been done under the Clinical Trial Registry of India with registration number CTRI/2022/03/041256 dated 22/03/2022. Patient accruals began in July 2022 and are projected to be concluded by July 2024. This protocol has been designed following the SPIRIT 2013 Statement.

### Study population

The cohort will be adults aged 18-80 years, reporting to the Head and Neck Surgery department of Amrita Institute of Medical Sciences Kochi (tertiary care centre) with histopathological confirmed oral tongue squamous cell carcinoma who has not undergone any other treatment other than diagnostic biopsy, planned for curative intent surgery with neck dissection.

### Eligibility criteria

Eligibility criteria are summarised in Table 1.

**Table 1.** Inclusion & Exclusion Criteria.

Inclusion criteria	Exclusion criteria
Age- 18-80 years OTSCC patient reporting to this center without any previous tumour treatment history other than diagnostic biopsy.	Other subsites of oral squamous cell carcinoma.
Curative intent surgery as a primary modality of treatment with neck dissection.	OTSCC indicative of only wide local excision (not indicative of neck dissection).
T 1 to T4 with and without nodal metastasis and willing to sign the Informed Consent.	Secondary primary tumors or recurrent tumours.
Patients who agree to complete the treatment as per standard treatment protocol and routine follow-up visits.	OTSCC patients on neoadjuvant chemotherapy.

### Study condition (oral tongue squamous cell carcinoma-OTSCC)

Oral carcinoma commonly known as oral squamous carcinoma (OSCC) occurs as an ulcero proliferative lesion affecting any site starting from the lips to the oropharynx. OTSCC is the most common OSCC and often initiates at the flat thin squamous cells that line the surface of the tongue. ^9^ The aggressive biological behaviour and clinically unpredictable prognosis of tongue cancer with close affinity to the vascular lymphatic network necessitate the need for further research in this area.

### Study implementation/methodology

Patient inclusion is done based on the inclusion criteria. The trial will enroll 97 eligible patients. After obtaining written informed consent, blood samples and tissue biopsy blocks will be collected from them.

For CTC/CTM evaluation – Blood samples with minimum trauma will be collected before and 6 months after surgery. 5 mL of peripheral whole blood samples will be collected in 10-mL vacutainer tubes (Becton Dickinson, New Jersey). The blood samples will be stored in the refrigerator at 2 to 8 ^0^ C. CTCs will be isolated by using Drugs Controller General of India (DCGI) approved OncoDiscover liquid biopsy technology. The OncoDiscover CTC isolation technique uses multifunctional, iron oxide-based, magneto-polymeric, and anti-epithelial cell adhesion molecule (EpCAM) targeting superparamagnetic nanoparticles. ^10^ Enumeration of CTC is based on CD45-, EpCAM+ and CK8,CK18 &CK19+ expression. CTM clusters are a group of two or more aggregated CTCs. The number of CTCs positive for PD-L1 expression is also evaluated.

### Immunohistochemistry (IHC) evaluation

**Figure 1.**
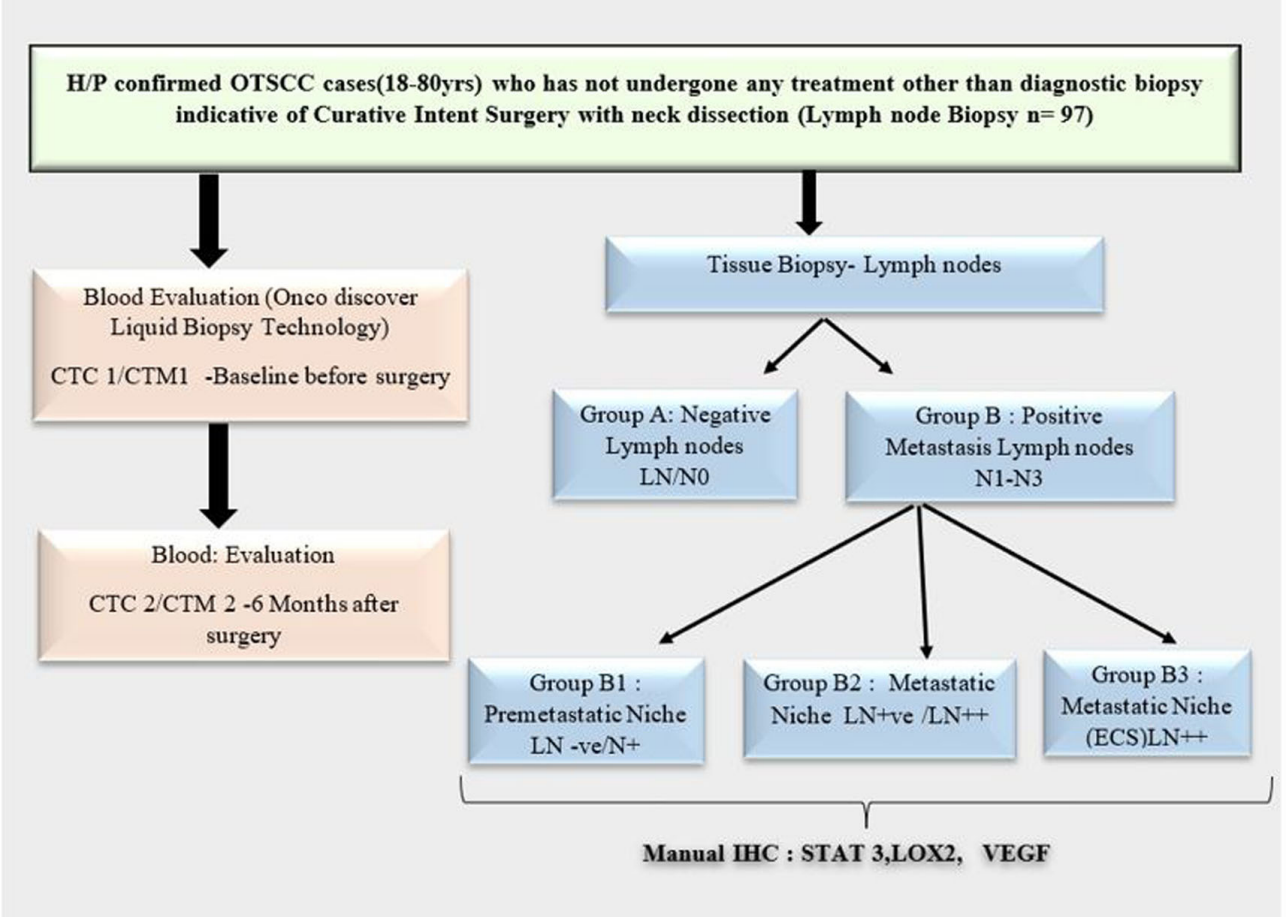
Trial Design of PMN Study.

Lymph nodes from these patients will be fixed and sectioned for routine histopathological evaluation. Formalin-fixed paraffin-embedded (FFPE) samples of the study may include T 1 to T4 with and without nodal metastasis. The lymph node samples will be grouped as Group A & B ( Figure 1) based on histopathological findings.

The lymph nodes collected for analysis will include nodes without disease both in groups, where there is no nodal disease (-ve LN in NO) and those with nodal disease (-ve LN in N+). Also, will include lymph nodes with disease (LN+) and those with ECS (LN++). The molecular markers studied in the lymph nodes will be STAT 3, LOX 2, and VEGF A. IHC technique to be employed with primary and secondary antibody standardization protocol.

Group A: First echelon lymph nodes will be sampled in pathologic N0 (-ve node in NO).

Group B: In node-positive cases, - a normal uninvolved lymph node is taken just distal to the level of lowest positivity ie. if level 2 is involved, an uninvolved node from level 3 is taken (B1). The lymph nodes with positive disease (B2) and those with extracapsular spread (B3) will be included in the analysis when present.

To investigate the molecular characteristic features in the formation of premetastatic lymph nodes, selecting nonmetastatic LNs in the vicinity of metastatic LNs seems to be most suitable. Being taken from the same patient most of the other variables can be avoided.

The preparation of slides will be done in accordance with standard IHC techniques. Manual IHC with antigen retrieval in pressure cooker will be done. All antibody standardization will be done according to the company protocol manual. All stained slides shall be photomicrographed within 48 hours. Interpretation will be performed independently by two pathologists who were blinded to the other parameters of the subject. In cases of disparity, a third pathologist will be consulted and the best concordant result will be accepted for analysis. IHC slides will be graded according to staining intensity. The patients will be followed up as per the standard of care ie 2 monthly for the first year and 3 monthly in the second year. Clinical evaluation supplemented by imaging if needed will be used during follow-up. The overall survival, recurrence-free survival, locoregional, and systemic failure rates will be determined at the end of two years follow-ups.

### Primary outcomes

• The correlation of molecular expression of STAT 3, LOX 2, and VEGF in the premetastatic nodes (-ve N) with recurrence -free survival. These markers will be correlated individually. This will allow us to analyse the significance of premetastatic niche formation and the pathways leading to it.
• To correlate the presence of CTC/CTM at two different time points to recurrence-free survival. This will allow us to establish the role of liquid biopsy in prognostication ie. the presence of CTC as a predictor/indicator of tumor spread via blood or lymphatic channels.
• The evaluation of Stat3 expression in tissue and PD- L1 in CTC will reveal on the local and systemic immune status of patients respectively. Those PD-L -1positive CTCs may be a strong indicator of those exceptional CTCs that escape the immune surveillance mechanism.

### Secondary outcomes

• The molecular markers for PMN will be compared in N0 and N+/N++ patients. This may allow a better understanding of the pathways of PMN formation. We expect to see the PMN changes be more prominent in the -ve N samples of N+/N++ patients.
• Any architectural histopathologic feature which correlates with the PMN changes if identified will also help as a predictive marker.
• To identify the role of CTC to predict PMN formation.

### Participation timeline

All subjects will be part of the trial for two years post initiation of the treatment or till the time when they show progressive incurable disease.

### Statistical analysis plan

*Sample size calculation*

The sample size is determined by the formula, n=(Z1−α22)pqd2 , z = Co-efficient of significance (1.96), α = Level of significance (5.0%), p = Prevalence of oral tongue tumour (50.0%) q = 1- p (50.0%), d = Desired precision (10.0%).

The minimum sample size for the study is computed and found to be 97 patients.

*Statistical method*

Chi-square Test to test the statistical significance of the association of the molecular expression (severity) of antibodies in lymph nodes and circulating tumor cells as numbers in oral tongue squamous cell carcinoma patients. To find the survival probability of disease-free survival, Kaplan Meier analysis and the comparison will be done using a log-rank test.

### Methodological issues

• The study investigates the molecular characteristics of the premetastatic, metastatic lymph nodes in OTSCC related to the most suitable and real control group.
• The CTC PMN association if present may help to correlate two triggering factors in overt metastasis formation.
• The primary limitation of this study is the limited number of antibodies used to identify the molecular PMN characteristics. The study is aiming to co-relate the molecular markers of different possible events in organ remodelling and immature pre-metastatic niche formation. However, to get a wholesome picture of PMN features a greater number of antibodies to be included for evaluation. Due to budgetary constraints, we have limited our molecular markers by choosing the best which describe the maximum possible molecular events. Also, the lack of sequential blood evaluation for CTC in more frequent intervals will not allow us to comment on its time of appearance and its effect on metastasis accurately.
  CTC evaluation is based only on EpCAM and cytokeratin without considering the Epithelial-Mesenchymal Transition (EMT) mechanism, stemness characteristics or its subpopulations.

### Data management

Site investigators will take up the responsibility for the conduct of the study. Project investigators are responsible for ensuring International Conference on Harmonisation Good Clinical Practice guidelines. Periodic review and data monitoring will be done by the University research team.

### Study status

The study is still in the recruiting phase. 27 patients have been recruited. In all these subjects the CTC sample has been evaluated at the pre-treatment time. The lymph node specimens after fixation have been collected as per protocol. The standardization of the IHC markers is ongoing after which the evaluation of the nodes will ensue. The patient follow-up is also progressing at the specified dates.

## Discussion

Metastasis can even occur many years after surgical resection of primary cancer due to tumor dormancy. Subramaniam N et al ^5^ on a study of OTSCC show 20-30% recurrence within 12 months and 40-50% mortality within 5year even after guideline-based treatment. This study showed younger patients had a higher incidence of tongue tumours with increased adverse pathological features. ^5^

In the study by Mizrachi et al. 15% of cN0 oral cancer patients developed neck recurrence. ^6^ Blatt et al. in their study on tumor recurrence among OSCC, which is one of the largest retrospective studies on oral squamous cell carcinoma described that recurrence is very frequent especially in the first six months after primary tumor diagnosis, they reported approximately 64% with local recurrences. ^7^

The new paradigms of metastatic biology research signify that metastasis is not a late onset event in tumor development nor related to tumor volume. Although progress has been made in understanding the mechanism of cancer spread, the complexity of the metastatic process remains a stumbling block. ^8^ Cancer cells are dynamic, with greater plasticity and can build their own niches. ^8^^,^^9^ Each cancer cell must be viewed as an organism capable of developing an entire tumour. ^8^ Metastasis is a process in which genetic instability of the primary tumor fuels cell heterogeneity, permitting cloning of a few metastatic cells that ultimately emerge and spread the tumour. ^9^^–^^11^

Tumor metastasis is now believed to be closely pursued by prometastatic milieu, premetastatic niche and metastatic niche formation. ^12^ Premetastatic niche is an area devoid of tumour cells, but it can nurture cancer cells. It provides a favourable microenvironment for tumour invasion, endurance and/or proliferation of malignant cells later to develop into metastasis. ^13^

These are noncancerous changes in a tumor-free organ and may be the most primitive suggestion of metastasis. Lymph nodes have been suggested to offer fertile soil for cancer cell seeding, proliferation, and metastasis. ^14^ These act as crucial metastatic spots and are a decisive prognostic parameter in diverse tumor types. ^15^^,^^16^

Primary tumor initiates the sentinel lymph node remodelling by releasing extracellular vesicles, soluble factors, a variety of cytokines, and growth factors before metastasis spread. ^11^^,^^17^

Recent studies have provided evidence on the critical role of primary tumour in tumor progression and metastatic spread. ^16^^,^^18^ Extra Cellular Matrix (ECM) remodelling is the key defining feature of PMN development. Fibronectin (FN), lysyl oxidase (LOX), bone marrow-derived cells (VEGFR-1), and matrix metalloproteinase (MMP)-9are key factors responsible for PMN initiation. The lysyl oxidase (LOX) family plays pivotal roles in PMN collagen remodelling, and thus in immune cell recruitment by ECM pre-conditioning. ^19^^,^^20^ Wakisaka N et al., in their study of OSCC clarified that tumour-draining sentinel lymph nodes showed greater lymphangiogenesis even much before cancer metastasis. It can function as a permissive "lymphatic niche" for tumour cell survival. ^21^

### Molecular characteristics of PMN: Antibody selection

**Figure 2.**
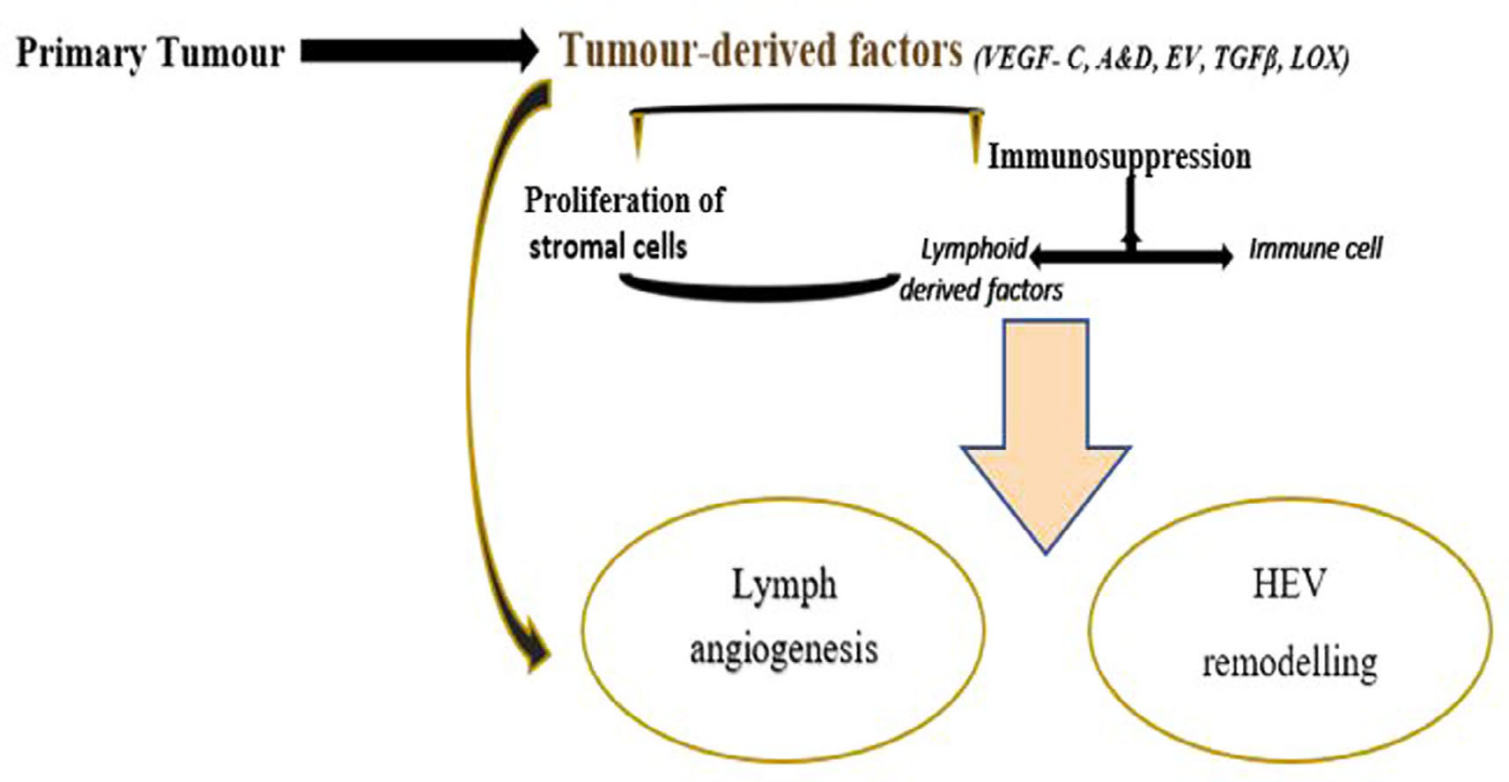
Schematic representation on molecular events on PMN formation in LN. ^12^^,^^19^

The primary tumor-derived secretory factors result in lymphangiogenesis and high endothelial venule (HEV) remodelling which are critical vascular events in PMN formation ^12^^,^^19^ ( Figure 2). Lymphangiogenesis in the premetastatic niche is a dynamic phase in tumor metastasis and lymphatic vessels may serve as a starting site for lymphatic dissemination of tumours.

Tumor-derived factors (VEGF, LOX, Tumour Growth Factors, extracellular vesicles) initiates immunosuppression by recruiting macrophages, MDSCs and regulatory T cells. ^18^^–^^20^ Though it has been widely accepted that far-reaching effects of cancer progression are achieved through immunosuppression, preliminary studies ^12^^,^^18^^,^^20^of PMN were focused on extracellular matrix modifications and stromal reprogramming.

Recruited myeloid cells are critical drivers of PMN formation and inflammation. PMN inflammation is shaped by the production of damage-associated molecular pattern (DAMP) molecules. DAMP recognition receptors induce potent STAT3- and NF-kB-mediated inflammatory signaling which regulates PMN myeloid cell composition and function. ^19^^,^^22^

STAT3 is a cytoplasmic transcription factor that regulates cell angiogenesis, inflammation, proliferation, differentiation, apoptosis, and immune response. ^22^ Recent studies have suggested that activated STAT 3 upregulates VEGF expression, thereby inducing tumour angiogenesis. STAT3 activation also increases immunosuppression activities. ^23^^‐^^25^

Wu LJ et al., on immunohistochemical analyses demonstrated that overexpression of STAT3 in tumor cell-free lymph nodes of gastric tumors was significantly associated with tumor recurrence. ^26^ They also revealed that persistent STAT3 activation in tumor-free lymph nodes was positively related to poor overall survival. ^26^ Several pre-clinical studies have suggested STAT3 inhibition may be a promising target for improving targeted cancer treatment. ^27^^,^^28^

LOXL2 has found in promoting lung pre-metastatic niche formation, there by lung metastasis and its pathological role in metastasis has been established. ^29^

LOX is a tumor-secreted protein increased in hypoxia and is found to be critically involved in premetastatic niche formation. LOX expression is associated with metastasis and poor survival in patients with breast or head and neck cancer. ^30^

Clinical data show that tumor-derived VEGF-A and VEGF-D generate lymphatic vessels before lymph node-induced metastasis, and are associated with lymph node metastasis. ^31^

A newly published literature showed that the tumor cells manage to infiltrate the lymph nodes by tricking the immune system to accept them as the body’s own cells. This gives tumor cells an easy entry for enabling metastasis. ^32^

STAT3 activation plays a major role in protecting the tumour cells from the body’s immune surveillance during their transit through circulation. ^33^ STAT3 is found to induce immunosuppression by upregulating PD-L1 in head and neck squamous cell carcinoma. STAT3 signalling activation was found to increase the probability of tumour cell survival, thus increasing the chances of invading distant organs potentially to form secondary tumour. ^33^^‐^^35^

Evolving evidence establishes a time-series event—the premetastatic niche has a reflective impact on cancer metastasis. ^19^^–^^20^^,^^36^ Tumor-promoting pre-metastatic changes in secondary organs may bring an unrecognized degree of complexity in curing metastatic disease. The critical machinery in PMN establishment is primary tumor-derived factors, exosomes, cell-free DNA (cfDNA), and circulating tumor cells. ^37^^,^^38^

### Rationale on the evaluation of circulating tumor cells

CTC characterizes microscopically disseminated disease which may have clinical implications. So, CTCs detection in node-negative patients may imply evidence of tumor cells that have escaped from lymphatic filtering and immunosurveillance mechanism. ^39^^,^^40^ Even though CTCs decrease during cascades of metastatic events but their self-seeding potential ^41^ is dangerous and can invade the primary tumour or progresses to clinically noticeable metastases. Blood vessels in tumors are abnormal, defective, and possess leaky endothelium. ^42^ This may influence the internal environment of tumors and perhaps the rate of angiogenesis. The loose vasculature and close anatomical access of the tongue may ease the re-entry of CTC to the primary site other than the potential risk of distant metastasis. ^43^^,^^44^ CTC detection in mouse models demonstrated that metastatic dissemination is not necessarily a unidirectional process. ^45^ Recent study demonstrated that lymphatic and haematogenous route can even occur together. The study demonstrated that tumour cells invade blood vessels within lymphnode and leaves the lymph node and enter circulation. ^45^^,^^46^

Metastatic progression is now presumed to be of linear and parallel models. ^47^^–^^49^ The genetic and epigenetic alterations within the primary tumor waves metastases in the linear progression model whereas in the parallel model, the preclinical distribution of less advanced disseminated tumor cells with self-regulating selection expands at the ectopic sites. ^47^^–^^49^ The presence of CTC can characterize both tumor progression models or even indicate lymph node skip metastasis.

Hristozova T et al. ^50^ suggested a strong correlation of CTC with regional metastasis in inoperable head and neck squamous cell carcinoma. Qayyumi et al. ^51^ found CTC as a poor prognostic factor in the overall survival of naïve OSCC patients in the Indian population. In their study, they found that pre-surgical CTC level has strong adversity on clinicopathological factors. They reported a positive correlation between CTC number and nodal metastasis. They concluded that 20.5% of clinically node-negative patients were pathological harboring metastasis.

CTCs may represent cells that are predisposed to the evolution of metastasis with friable intercellular connections. CTCs symbolize a biologically aggressive tumor with higher versatility to evade the immune surveillance mechanism. ^52^ These characteristics of CTCs may provide a valuable tool to detect this clinical subgroup and guide systemic therapies in a more individualized manner. ^52^

The survival of CTCs is subjected to their ability to withstand various nonspecific forces, the turbulence of circulation etc. ^52^^,^^53^ So, a very low percentage of tumor cells survive which further establishes micro metastasis in distant organs. ^54^

Circulating tumor microemboli not always a mere tumor cell cluster but it may exhibit varying phenotypic and molecular characteristics than single CTCs. These may provide intuitions into the heterogeneity and biological behaviour of tumour. ^55^ CTM metastases by cell jamming that produces homotypic monoclonal or polyclonal tumor clusters. These cells can interact with stromal or immune cells in the inflammatory peri-tumoral infiltrate forming heterotypic clusters. Clustering of CTC can withstand shear stress resistance and enhances their stemness with increased metastatic potential. ^56^

Although studies on the clinical significance of PD-L1-positive (PD-L1 ^+^) CTCs in head and neck cancers are in their infancy, PD-L 1 positive CTC has clinical relevance in many other solid cancers. PD L 1 may be upregulated in CTCs undergoing EMT and its expression is correlated with poor survival and therapy resistance. ^57^ Some recent works have shown that it could be a prognostic biomarker in renal, epithelial ovarian and lung, advanced urothelial and metastatic breast malignancies. ^57^^–^^59^

Tissue biopsy fails to reveal on the spatiotemporal heterogenicity and its expression is tissue may not be always adequate, thus it could also help to predict the anti PD L 1 or targeted therapy responses. ^58^ The expression of PD-L1 on circulating tumor cells may also be a reliable predictive biomarker. ^60^

PD -L 1 expression in conventional immunohistochemistry assays lacks accuracy and reliability as the staining of cytoplasmic proteins interferes with cell membrane protein estimation. ^61^

As discussed earlier, STAT3 activation increases immunosuppression and found to induce immunosuppression by upregulating PD-L1 in head and neck squamous cell carcinoma. Concordance in local immunosuppression by STAT 3 tissue molecular profiling and PD L 1 on CTC/liquid biopsy for systemic immunosuppression will be interesting to look at, which is also being done in this study.

The utility of CTCs in the diagnosis of early-stage cancers are least explored because CTCs were initially believed to be a feature of advanced-stage disease. ^62^^,^^63^ More clinical trials to look at the pre- and postsurgical CTC counts in the same patient may be necessary for therapeutic implications. ^63^ Evolving evidences specifies that CTCs detecting at early stages are indicating the development of aggressive cancers. ^64^ Therefore, this study may have great potential to be used for early cancer detection as well as avoiding overdiagnosis of indolent disease. This study also opens windows to the budding concept of cancer metastasis chemoprevention.

***Ethics &dissemination***

The trial will be conducted by the principles of the Declaration of Helsinki and guidelines of the Indian Council of Medical Research. The protocol has been approved by the Ethics Committee Amrita School of Medicine (AIMS), Kochi India (ECASM-AIMS-2022-048). The principal investigator will submit an Annual Progress report throughout the clinical trial or as on request. The final report along with trial end notification will also be submitted. No unauthorized persons will have access to any data about this trail. Patients will be educated about the trial which will detail the exact nature of the trial, implications, and constraints, followed by which printed information sheets will be given. Informed consent will be taken and documented.

## Conclusion

Premetastatic niche formation has been found to have a role in defining tumor progression. The present study will help to elucidate their significance in oral cancers,which is less studied. It may be difficult to histologically assess the PMN changes in other metastatic prone organs like liver or lung, but changes in the lymph nodes can be easily evaluated. So, these changes in lymph node and the correlation with circulating tumor cells may act as an indicator of both regional as well as distant metastasis, thus delineate better targets for therapy.

## Data Availability

No data are associated with this article.
