# Supplementary material for: Evaluation of  premetastatic changes in lymph nodes(pN0) of oral tongue tumour: A prospective observational Study
Source: F1000Res. 2023 Jul 26;12:889. [Version 1] doi: 10.12688/f1000research.138951.1 (PMC10541534; doi:10.12688/f1000research.138951.1)
Supplement: Peer review history

# Reviewer report: Reviewer response for version 1

**DOI:** 10.5256/f1000research.152188.r204183

**Published:** 2023-09-29

**Authors:** Pankaj Chaturvedi

**Affiliations:** 1 Department of Surgical Oncology, Tata Memorial Centre, Homi Bhabha National Institute, Mumbai, Maharashtra, India

Tongue cancers have high risk of regional failure even after receiving standard treatment protocols. There is unmet need for detecting the premetastatic changes which can predict regional or distant failures. However, the major caveat in detecting these premetastatic markers is the heterogeneity of tumor, the impact of heterogeneity in the development of LN metastasis, and multiple mechanisms involved ^1^.

 Lymph node lymphangiogenesis (LNL) is one of the most distinctive aspects of premetastatic changes and thus a strong predictor of lymph node metastasis. However, LNL is a complex process modulated by various growth factors and pathways ^2^. ^^VEGF-C, VEGF-D, and VEGF-A are some of the most researched components for lymphangiogenesis ^3^. Their elevated levels are associated with LN and distant metastasis ^4^^,^^5^. Several tyrosine kinase receptors are targeted by receptor tyrosine kinase inhibitors, however, the exact pathway is unknown ^6^. The S1PR1-STAT3 pathway enables myeloid cells to intravasate, turning the organ microenvironment conducive for proliferation, survival, and initiation of metastasis ^7^. LOX expression is mainly in the tumor cells which are exposed to relevant levels of hypoxia. LOX is an important component of pre-metastatic niche formation and metastasis ^8^. Even though there are no standard treatment available, there is promising preliminary data regarding therapeutic targeting of STAT3 and LOX ^9^^,^^10^.

 Circulating tumor cells (CTCs) are rare cells that escape from the primary tumor and enter into the bloodstream to form metastatic deposits. Elevated CTC levels have a strong correlation with adverse survival outcomes ^11^. Even though, it is a difficult task to establish a permanent in vitro cell line to conduct experiments ^12^. The CTCs have the potential for personalized treatment due to the possibility of individualized drug susceptibility testing ^13^.

 Several growth hormones, RNA, cytoplasmic proteins, and growth factors like Periostin, VEGF, Angiopoietins, Laminin γ2, and Insulin-like growth factor (IGF)-1/2 which are involved in the induction of lymphangiogenesis. Moreover the activation of mTOR, podoplanin, and lymphatic vessel endothelial hyaluronan receptor 1(LYVE-1), extracellular vesicle, and HPV +ve tumors may affect the lymphangiogenesis ^6^.

 The objectives of the present protocol which are to evaluate the correlation of molecular expression of STAT 3, LOX 2, and VEGF in the tumor-free lymph nodes (N0) to assess the premetastatic niche changes and recurrence-free survival and to evaluate the efficacy of CTC (circulating tumor cells) and CTM (circulating tumor microemboli) in terms of relapse, and disease-free survival in oral tongue squamous cell carcinoma patients is quite relevant . Moreover, the study design and methodology is described in detail and seems appropriate to achieve the above mentioned objectives.

 The admirable part of the present study is that it aims to include the various mechanisms of LN pre-metastatic niche creation in HNSCC, which are dependent on lymphangiogenesis, activation of the S1PR1-STAT3 pathway, LOX pathway, and CTCs. However, paying attention to other molecular pathways of lymphangiogenesis is suggested as a single molecular pathway is likely to lead to failure in terms of therapeutic development. Further research is needed to elucidate the optimal therapeutic molecular target(s) and explore newer HNSCC treatments, which can support new strategies for the patients.

 The absence of any previous study or information regarding the architectural histopathologic features to correlate with PMN changes makes it ambiguous. However, an appropriate description of these changes and photographic documentation might provide important information.

Is the study design appropriate for the research question?

Yes

Is the rationale for, and objectives of, the study clearly described?

Yes

Are sufficient details of the methods provided to allow replication by others?

Yes

Are the datasets clearly presented in a useable and accessible format?

Yes

Reviewer Expertise:

Head & Neck Oncology, Circulating tumour cells, molecular markers

I confirm that I have read this submission and believe that I have an appropriate level of expertise to confirm that it is of an acceptable scientific standard.

## References

[1] : Intra-tumour heterogeneity: a looking glass for cancer?. *Nat Rev Cancer* .2012;12(5) : 10.1038/nrc3261 323-34 10.1038/nrc3261 22513401

[2] : The lymph node pre-metastatic niche. *J Mol Med (Berl)* .2015;93(11) : 10.1007/s00109-015-1351-6 1173-84 10.1007/s00109-015-1351-6 26489604

[3] : Cancer stem cells, lymphangiogenesis, and lymphatic metastasis. *Cancer Lett* .2015;357(2) : 10.1016/j.canlet.2014.12.013 438-47 10.1016/j.canlet.2014.12.013 25497008

[4] : VEGF-C and VEGF-D expression is correlated with lymphatic vessel density and lymph node metastasis in oral squamous cell carcinoma: Implications for use as a prognostic marker. *Int J Oncol* .2009;34(3) : 10.3892/ijo_00000193 673-80 10.3892/ijo_00000193 19212672

[5] : Head and neck squamous cell carcinoma lymphatic spread and survival: Relevance of vascular endothelial growth factor family for tumor evaluation. *Head Neck* .2015;37(10) : 10.1002/hed.23765 1410-6 10.1002/hed.23765 24824527

[6] : The Regulation of Lymph Node Pre-Metastatic Niche Formation in Head and Neck Squamous Cell Carcinoma. *Front Oncol* .2022;12: 10.3389/fonc.2022.852611 852611 10.3389/fonc.2022.852611 35574333PMC9094482

[7] : The pathogenesis of cancer metastasis: the 'seed and soil' hypothesis revisited. *Nat Rev Cancer* .2003;3(6) : 10.1038/nrc1098 453-8 10.1038/nrc1098 12778135

[8] : Investigating hypoxic tumor physiology through gene expression patterns. *Oncogene* .2003;22(37) : 10.1038/sj.onc.1206703 5907-14 10.1038/sj.onc.1206703 12947397

[9] : A human colonic commensal promotes colon tumorigenesis via activation of T helper type 17 T cell responses. *Nat Med* .2009;15(9) : 10.1038/nm.2015 1016-22 10.1038/nm.2015 19701202PMC3034219

[10] : Tumor-associated Endo180 requires stromal-derived LOX to promote metastatic prostate cancer cell migration on human ECM surfaces. *Clin Exp Metastasis* .2016;33(2) : 10.1007/s10585-015-9765-7 151-65 10.1007/s10585-015-9765-7 26567111PMC4761374

[11] : Circulating tumor cells as a predictor for poor prognostic factors and overall survival in treatment naïve oral squamous cell carcinoma patients. *Oral Surg Oral Med Oral Pathol Oral Radiol* .2022;134(1) : 10.1016/j.oooo.2022.02.018 73-83 10.1016/j.oooo.2022.02.018 35595620

[12] : Cancer therapy. Ex vivo culture of circulating breast tumor cells for individualized testing of drug susceptibility. *Science* .2014;345(6193) : 10.1126/science.1253533 216-20 10.1126/science.1253533 25013076PMC4358808

[13] : Circulating tumor cells as a promising target for individualized drug susceptibility tests in cancer therapy. *Biochem Pharmacol* .2021;188: 10.1016/j.bcp.2021.114589 114589 10.1016/j.bcp.2021.114589 33932470

# Reviewer report: Reviewer response for version 1

**DOI:** 10.5256/f1000research.152188.r190856

**Published:** 2023-08-14

**Authors:** Maya Ramesh

**Affiliations:** 1 Department of Oral Pathology, Department of Pedodontics, Vinayaka Mission’s Sankarachariyar Dental College, Salem, Tamil Nadu, India

The study goal is to find the premetastatic changes in oral tongue carcinoma which are very common in India. So, the study is relevant in the Indian scenario which can provide unsurpassed outcomes. The study methodology is good as it uses liquid as well as tissue biopsy. The schematic representation is fully describing the methodology but more narrative on lymph node sampling is better appreciated. Only very few studies have been done in oral cancers with liquid biopsy.

 Circulating tumor cells have been implicated in loco-regional and distant metastasis and their role is head and neck region ^1^ is found but lack of evidence in tongue tumor. PDL-1 can represent the immune status of the patients and it is found to be a good biomarker ^2^. So, how far premetastatic changes can be addressed with liquid biopsy in tongue tumors has to be proved and at present lacks high scientific representation.

 Immunohistochemistry – More number of antibodies might have been considered as clearly mentioned in methodological issues.

 The authors have well illustrated the rationale with supporting literature, so if the rationale can be achieved it is a good protocol that can be replicated by the scientific community.

Is the study design appropriate for the research question?

Yes

Is the rationale for, and objectives of, the study clearly described?

Yes

Are sufficient details of the methods provided to allow replication by others?

Yes

Are the datasets clearly presented in a useable and accessible format?

Partly

Reviewer Expertise:

Immunohistochemical study in Oral Cancer, dental fluorosis

I confirm that I have read this submission and believe that I have an appropriate level of expertise to confirm that it is of an acceptable scientific standard.

## References

[1] : Circulating tumour cells in head and neck cancers: Biological insights. *J Oral Pathol Med* .2020;49(9) : 10.1111/jop.13075 842-848 10.1111/jop.13075 32526815

[2] : Circulating Tumour Cell Expression of Immune Markers as Prognostic and Therapeutic Biomarkers in Head and Neck Squamous Cell Carcinoma: A Systematic Review and Meta-Analysis. *Int J Mol Sci* .2020;21(21) : 10.3390/ijms21218229 10.3390/ijms21218229 33153130PMC7662307
